# Supplementary material for: Characterization of Iflavirus in the Red Flour Beetle, Tribolium castaneum (Coleoptera; Tenebrionidae)
Source: Insects. 2023 Feb 23;14(3):220. doi: 10.3390/insects14030220 (PMC10051554; doi:10.3390/insects14030220)
Supplement: Supplementary file 1 [file insects-14-00220-s001.zip › Supplementary data S1. Populations tested for TcIV prevalence.pdf]

Fatehi et al. 2023. Characterization of Iflavirus in the Red Flour Beetle, *Tribolium Castaneum* (Tenebrionidae; Coleoptera).

Supplementary data S1. *Tribolium castaneum* strains used for TcIV prevalence study

**Species/Population**	**Original collection**	**Rearing Laboratory**	**TcIV**
* USDA	Unknown	Phillips	-
Columbia-1	Columbia, MO	Phillips	-
Japan-4	Ibaraki prefecture, Japan	Phillips	-
Brazil-4	Brazil	Phillips	-
Z-2	Chickasha, OK	Phillips	-
Z-4	Cedar Rapids, IA	Phillips	-
Abilene-1	Abilene, KS	Phillips	-
NDG-2	Winnipeg, Canada	Phillips	-
Costa Rica-1	Costa Rica	Phillips	-
Hudson, KS	Hudson, KS	Phillips	-
Brazil	Brazil	Phillips	-
Thailand	Thailand	Phillips	-
CO, Pyr-R	Unknown	Phillips	-
Brazil-5	Pacheco, Brazil	Phillips	-
Shellenberger	Manhattan, KS	Phillips	-
A20 Rdiel	England	Phillips	-
GA2	Georgia	Phillips	+
Kansas-1	KS	Phillips	+
Belle Glade, FL	Belle Glade, FL	Phillips	+
Winnipeg, MB	Winnipeg, Canada	Phillips	+
Dark Shell`	Manhattan, KS	Phillips	+
Parlier, CA	Parlier, CA	Phillips	+
Rdiel-Bcy-Lab-S	Unknown	Phillips	+
GA1	Georgia	Phillips	+
Red Level	Alabama	Phillips	+
Goliath	Derived from GA1	Phillips	+
Arbuckle, CA	Arbuckle, CA	Phillips	+
Myrtle Grove, LA	Los Angeles, CA	Phillips	+
Nashville, GA	Nashville, GA	Phillips	+
Jonesboro, AR	Jonesboro, AR	Phillips	+
California	CA	Phillips	+
Stuttgart, AR	Stuttgart, AR	Phillips	+
Lab-S-Rusty	USDA	Phillips	+
Sokoloff-8	Chicago	Phillips	+
QTC-279-Pyr-R	Australia	Phillips	+
Ozark, AL	Ozark, AL	Phillips	+
GA1	Georgia	Zhu	+
GA1	Georgia	Brown	+
GA2	Georgia	Brown	+
Goliath	Derived from GA1	Park	+
GA1	Georgia	Park	+
Vermillion	Derived from GA1	Park	+
Goliath×GA1	Derived from GA1	Park	+
Tribolium freemani	Manhattan, KS	Park	-
Tribolium confusum	Belgium	Phillips	-

Tribolium brevicornis	USDA-ARS, Manhattan, KS	Scully	-
Palorus ratzeburgii	USDA-ARS, Manhattan, KS	Scully	-
Cynaeus angustus	USDA-ARS, Manhattan, KS	Scully	-
Gnathocerus cornutus	USDA-ARS, Manhattan, KS	Scully	-
Latheticus oryzae	USDA-ARS, Manhattan, KS	Scully	-

* USDA-ARS: Center for Grain and Animal Health Research, Manhattan, KS, USA
