# Supplementary material for: Characterization of Iflavirus in the Red Flour Beetle, Tribolium castaneum (Coleoptera; Tenebrionidae)
Source: Insects. 2023 Feb 23;14(3):220. doi: 10.3390/insects14030220 (PMC10051554; doi:10.3390/insects14030220)
Supplement: Supplementary file 1 [file insects-14-00220-s001.zip › Supplementary data S3. Oligonucleotides used for qPCR.pdf]

Fatehi et al. 2023. Characterization of Iflavirus in the Red Flour Beetle, *Tribolium Castaneum* (Tenebrionidae; Coleoptera).

Supplementary data S3. The oligonucleotides used for qPCR

**Primer**	**Sequence**
RdRp-RT-F	CCCTTGAGAGTTTTAATGCTCATGTG
RdRp-Probe	CTCAAGAACCACACTTCACGAATATCGGT
RdRp-RT-R	CGCCGCATTCTGGATACTATC
rpS3-F	GACCGTGTTTTGGGGGAGAAGA
rpS3-R	CTTTAGCGCCACATTCCATGATGT
rpS3-Probe	CTAAGAGACTCTGCTTGTGCAATGGCA
