# Supplementary material for: Characterization of Iflavirus in the Red Flour Beetle, Tribolium castaneum (Coleoptera; Tenebrionidae)
Source: Insects. 2023 Feb 23;14(3):220. doi: 10.3390/insects14030220 (PMC10051554; doi:10.3390/insects14030220)
Supplement: Supplementary file 1 [file insects-14-00220-s001.zip › Supplementary data S4. Sequence alignment of iflaviruses, TcIV, DWV, SBPV, and DsLJV.pdf]

Fatehi et al. 2023. Characterization of Iflavirus in the Red Flour Beetle, *Tribolium Castaneum* (Tenebrionidae; Coleoptera).

Supplementary data S4. Sequence alignment iflaviruses, TcIV, DWV, SBPV, and DsLJV

```
AUE23905 TcIV     1    -MESLSSNVSFVQEVKALFKKQKLVINLAAENAEMKSLRETRKI-YASCD
ATG71380 DWV      1    -MAFSCGTLSY------------------SAVAQAPSVAHAPRT-WEVDE
YP_003622540 SBPV 1    -MEHTPLLSSF-------------------PITLLDHCGGNRKIHWTRCY
QVD33034 DsLJV    1    MQSFVEGIHNMEQ----------------LDNGRIKRSKHIPKGFKPICN

AUE23905 TcIV     49   DDLLQSILRFPSRKAALDHVDLEILKLRFKINAKLHSCSSKIDKVLYWFF
ATG71380 DWV      31   A----------RRRRVIKRLALEQERMRNVLDADVY--------------
YP_003622540 SBPV 31   E-------------------------------------------------
QVD33034 DsLJV    35   R-------------------------------------------------

AUE23905 TcIV     99   VNDKLDQLDLFKHPVTRNNLPYCQFRVKQFLA---IRKKYCKKTYDISEQ
ATG71380 DWV      56   ---------------YQATWEQEDARDNEFLM---EQLNNLYTIYSIAER
YP_003622540 SBPV 31   ---------------QKYWLPFSCCKVASRLPCVNVRRSYMWYSKKQSKW
QVD33034 DsLJV    35   --------------------QYETCKDES-------GRGYINISFKPLDN

AUE23905 TcIV     146  YLYNQEMNARKYSKKQKSFVPTKQYYTRVPLSSVS--SSINRFEVLENIE
ATG71380 DWV      88   -------------------------CTRRPIKECSPISVSNRFAPLESLK
YP_003622540 SBPV 66   -----------------------IYLSESDFRACK-AGIYKRREEQEKEK
QVD33034 DsLJV    58   -------------------------TDSRETLACALERFARLRLKRKSNR

AUE23905 TcIV     194  LPEPQQDSIIEDDPMYRFHAALFRNGQEISVQLGRHRLDYSNVSYDVQRP
ATG71380 DWV      114  VEVGQEAS-----------ECMFKKPKYTRVCKKVKRVTTRFVREKVVRP
YP_003622540 SBPV 93   L--------------------------WSELCDICSWECFEYYKFRDQR-
QVD33034 DsLJV    84   VNRPNLTALFND---------------WVCGVTILEKQTSRAQERHNQQG

AUE23905 TcIV     244  YQPTVIPRPVDLWGRLRERLAALRELRLPTVFSPFKMFKNWFMPRDNYFE
ATG71380 DWV      152  ----MCSRSPMLLFKLKKIIYDLHLYRLRKQIRLLR--------------
YP_003622540 SBPV 115  -----------LLLLLRKKIADKAQCRCRTNCKLVTIKHGYVR-------
QVD33034 DsLJV    119  DRTRYVERNTYVLQFMDGML------RVNCSFKSFK-----FR-------

AUE23905 TcIV     294  AWIKFHIKEGHQE-YEMFVI-------DPCVEALPEMDT--ADAHGEGTL
ATG71380 DWV      184  -------RQKQRE-YELECVTSRLQLSDPVQAK-PEMDNPNPGPDGEGEV
YP_003622540 SBPV 147  -------RVKTIEPCEAIELTNAETFGSNLDFAQPEMDR--PEGSEERTV
QVD33034 DsLJV    150  -------NLQEII-YEAIIM-------ELLSIPQPQMED--DGGQGDTTQ
                                                 (LP-VP2 cleavage site)
AUE23905 TcIV     334  QTSQASNVVLTENREDSREVTADFQVTTWKDLCSSEFAANYDVNVDRWIL
ATG71380 DWV      226  ELEKDSNVVLTTQRDPSTSIPAPVSV-KWSRWTSNDVVDDYATITSRWYQ
YP_003622540 SBPV 189  QT---SNVVLGETNIESQDIASKEYSPTWDRLASSEVSDEYPMLTDRWLF
QVD33034 DsLJV    184  DSDKAGNTIITRDADQSVAKEEGIAIPQLISICSTEPMHQFESITNRWMA

AUE23905 TcIV     384  INLFEWTTAS-TQGALLKSYYLP---LHMVKKEVVTP--CRMPNTLPFTI
ATG71380 DWV      275  IAEFVWSKDD-PFDKELARLILPRALLSSIEANSDAI--CDVPNTIPFKV
YP_003622540 SBPV 236  WKSVKWEVNDSAFGKMLVQEKFPQSWVQMDVNVNNIPRYTNIPNFIPFNI
QVD33034 DsLJV    234  LPSIEVKTSD-AFNTILQTYNLP----SFLYSENTAP------NMMPFEN

AUE23905 TcIV     428  HRYWRGDLEIKIHINANKFQIGSLQCSWYYSPNLDAKIDKRLNLWTQSQA
ATG71380 DWV      322  HAYWRGDMEVRVQINSNKFQVGQLQATWYYSDHENLNISSKRSVYGFSQM
YP_003622540 SBPV 286  HQYMRADFEVKIYVNPNDFVSGWLIMAFLYQGSEMFDYKLRRNPAALMQM
QVD33034 DsLJV    273  FMFGKYDIWFKFVVNANKFHVGKVLASVKYDSYQID--TLRNSLPALVSR

AUE23905 TcIV     478  HHVIINASTSNEVTMVVPYKYHLPYLHTK--PRGDM----PSPPLNLGTL
ATG71380 DWV      372  DHALISASASNEAKLVIPFKHVYPFLPTRIVP--DW----TTGILDMGAL
YP_003622540 SBPV 336  PHVLVNVGAANEATLKIPYRYVRPFMRCKDILRGDNLITGVTEPLNMGVL
QVD33034 DsLJV    321  PHVMMDLAANNEGMLCVPFKYHRTFVRNASLANSQY----GSKAAQYASV

AUE23905 TcIV     522  YIRVLNPLTTSV--NGPTRANVSVFVRSFNNEFTGMLSGDIDKI-EDMPY
ATG71380 DWV      416  NIRVIAPLRMSA--TGPTTCNVVVFIKLNNSEFTGTSSGKFYAS-QIRAK
YP_003622540 SBPV 386  FVEVLIPFRTSAASSAPKSLDVSLFVKMTNAKFTGMVDGSIALLSKPIAL
```

```
QVD33034 DsLJV     367  VLSIMSPLAAAV--GAPSNMYIRPFYAIKKASFTGMSY-KIPVV-QMETA

AUE23905 TcIV      569  PEMDRILDVTAGVLNMLHPDNNRDNPPNPRPPQMLVPTASHSWSVGTGMA
ATG71380 DWV       463  PEMDRILNLAEGLLNNTIGGNNMDNPSYQQSPRHFVPTGMHSLALGTNLV
YP_003622540 SBPV 436  PEMDRILDNGLGVVSKLLKDVNCDNPPDPTPAKFFVPIPSHSWAHGTNTS
QVD33034 DsLJV     413  GAVVSMLDKGLKICGAI---KNMDKPYDDQRVQQVILRPRMNFQTGVGAS
              (VP2-VP4 cleavage site)    (VP4-VP3 cleavage site)
AUE23905 TcIV      619  EPIHPLRLDGRGQTPHPPGIE-VNNEMEIRTISRVFGLLETITWTNNHL-
ATG71380 DWV       513  EPLHALRLDAAGTTQHPVGCA-PDEDMTVSSIASRYGLIRQVQWKKDHA-
YP_003622540 SBPV 486  EPTNTLRLDG-GVVGVGRSDDIGTSDTAISGIIGVYGLLKPFDWNANDTG
QVD33034 DsLJV     460  DSV-PLRLDPLAQTTYLPDHEYPDDPTTVLDIAKIWGFAGAFTWKASST-

AUE23905 TcIV      666  ---QQQVIWSADASPFPRKNCHYTQPESDTSLGTYAIPPVGVLSSMFYGW
ATG71380 DWV       560  ---KGSLLLQLDADPFVQQRIEGTNPIS-----LYWFAPVGVVSSMFMQW
YP_003622540 SBPV 535  RNVGGHLLWSMPVHPQV-DKDQVIQVMTQSKLTQYYLPPISVVSSLYAYT
QVD33034 DsLJV     507  ---EGSELFNIPIEPTWRNRDSWAGVPT----------PLEYISSMYQFW

AUE23905 TcIV      714  RGTIEFRFDVVASQFHTGRLIIAYIPGVTSTTP--ISFAQLRASTNMVFS
ATG71380 DWV       603  RGSLEYRFDIIASQFHTGRLIVGYVPGLTASLRQQMDYMKLKSSSYVVFD
YP_003622540 SBPV 584  RGSIKYKFLFGNNPRHNARLLVAYIPGISSDNR--LTLERARNSAHVVFS
QVD33034 DsLJV     545  SGPLEVRLDFVSNAFHTGSIMLSAEFGRAST-----NVEQSGSTYTKVFH

AUE23905 TcIV      762  LQETDQFTFKVPFICNKPYWPREFAGDY--DRQNSAAPSR----------
ATG71380 DWV       653  LQESNSFTFEVPYVSYRPWWVRKYGGNY--LPSSTDAPST----------
YP_003622540 SBPV 632  LNEVSEFVFTVPYITDTMWWPRKYGGPQ--AAGEFVAPSY----------
QVD33034 DsLJV     590  LGNQKTVSFVIPYIYDTV-WRRTSTVPFSVLRTNLDASARSGQISTMRAN

AUE23905 TcIV      799  ---IYIAVLNQLIPMESVSNQVYINVYMRAGDDFELIVPVQPSIGLPYNT
ATG71380 DWV       690  ---LFMYVQVPLIPMEAVSDTIDINVYVRGGSSFEVCVPVQPSLGLNWNT
YP_003622540 SBPV 669  ---ICMFILNPLVAMESVPSIVTIVPMIAAGDDFEVAVPAQPAVGLSRNI
QVD33034 DsLJV     639  SSAFKVRVINKLVPVQSVVQDIQVLVFIRAADTFTLHSPIMSNM---LNS

AUE23905 TcIV      847  KYLVTNNIKTQIVALSGYYPYYSGTFEDFTEAQ--VFRWGTAAHQIAH-F
ATG71380 DWV       738  DFILRND--EEYRAKTGYAPYYAGVWHSFNNSNSLVFRWGSASDQIAQ-W
YP_003622540 SBPV 717  DVIYPKD--SIISFKSGYFPVYVGSWHSFFDSTKAILRYGAVSDHIAQ--
QVD33034 DsLJV     686  EQVYA------LQDFPGNYPLENVSELSKISLTDSVREPGAV--QVSHKW

AUE23905 TcIV      894  DDYNHSDTV-RATICTTTDTSLKYTDDK-GTQQTITWAV---------FF
ATG71380 DWV       785  PTISVP----RGELAF-----LRIRDGKQAAVGTQPWRT---------MV
YP_003622540 SBPV 762  -LGNIPANVNRKAFWIVVGDTIKFKT-KLDKINGTEWFIPEGEYTLGYGV
QVD33034 DsLJV     728  MKGQTPDQI-QNAILVTNEGTILSSNPDHPLIDPKNVSTAPDSKIESGII

AUE23905 TcIV      933  NLPQ--LGYFVGIP-CRSQAAATQLAINCQLKKKALNDPENTPYVLSNKW
ATG71380 DWV       817  VWPSG-HGYNIGIP-TYNAERARQLAQHLYGGGSLTDEKAKQLFVPANQ-
YP_003622540 SBPV 811  VWRDGAYAYMVPYPLTPLGEKIAQYTASLLASNTAISQ--IRPYIPDYI-
QVD33034 DsLJV     777  AAPNPASKYFWV---NSQGSRIT------YNPSILPPDDYQHVVLPDFE-

AUE23905 TcIV      980  STSSNTYCTGNPVWTVYAVQPNTL------------------AYAEGERG
ATG71380 DWV       863  -QGPGKVSNGNPVWEVMRAPLATQRAHVQDFE-----FID--AIPEGEES
YP_003622540 SBPV 857  --VDSAASKDNILWSPIEDRLRAQTE-----------WVM--AEPEMERT
QVD33034 DsLJV     816  ------AKKDAYYWNTARTRRYRYNRTYRTFDIGGGVWMDQPAFAQMDDG
                                                  (VP3-VP1 cleavage site)
AUE23905 TcIV      1012 QAADLINIGSV-ASTMQGLSIYGENFFSLKDLSRRYQLY--GVYNVTTFT
ATG71380 DWV       906  RNTTVLDTTTILQSSGFGRAFFGEAFNDLKTLMRRYQLY--GQLLLSVTT
YP_003622540 SBPV 893  FTPNVMQPTPLLPTTNDGRVTFGEAFNDLKDLARRYQLYWEGTILEGNLR
QVD33034 DsLJV     861  VKEDTDPTETFQIGTNRNNIQTVESHIRIKDILRR-PVCIVSHFNIPAYR

AUE23905 TcIV      1059 TSTQEYVK----IRFPAVPQGLALNIGTSANINEIFN--RCREGHIPIIA
ATG71380 DWV       954  DKNIDHCM----FTFPCLPQGLALDIGSAGSPHEIFN--RCRDGIIPLIA
YP_003622540 SBPV 943  AIRRNSAL----VQLPLYPHGLRIQPDVN---NPIWN--IMRDGHIPVIS
```

Fatehi et al. 2023. Characterization of Iflavirus in the Red Flour Beetle, *Tribolium Castaneum* (Tenebrionidae; Coleoptera).

```
QVD33034 DsLJV    910  NVAAGATLSVNPFFVPCLPPSHMITYGTSA--NRIFTPLIGRSAHTHLLD

AUE23905 TcIV     1103 SGYRFYRGSIRFRLICDPKIASMIVVQHRPDRRLRYLNIQSQDKTDI--D
ATG71380 DWV      998  SGYRFYRGDLRYKIVFPSNVNSNIWVQHRPDRRLEGWSAAKIINCDAVST
YP_003622540 SBPV 984  SGFRYFRGGLRLRIVVEG-LNSCVWVQHHPDRPSIF---SRPIIGRYIAA
QVD33034 DsLJV    958  L-FRFWRGSQRYSIISHRITGAPIYAVYVPHSGAMNCGTVTFLTVDLLDT

AUE23905 TcIV     1151 GDSVMNHTYASYIQLTRINGVIEFEVPYYQLGMYGLLQYPNVNEASDVGN
ATG71380 DWV      1048 GQGVYNHGYASHIQITRVNNVIELEVPFYNATCYTYLQAFNASSAAS--S
YP_003622540 SBPV 1030 KDAYRNHAYAAYVQNMSVNRTIEVEVPFYQPGLYGMLNASDNNTANSFDR
QVD33034 DsLJV    1007 NCPPSSFGLATEVMIPSVNPTMSIEVPYETENNWTLMQCENWER-----N

AUE23905 TcIV     1201 Y-YS-LGEIAVSMTMNTADIKSFKNSLLSIYYCMADDMSFSTFQGFPPMI
ATG71380 DWV      1096 YAVS-LGEISVGFQATSDDIATIVNKPVTIYYSIGDGMQFSQWVGYQPMM
YP_003622540 SBPV 1080 LRFTGLGDLLIGIEGEQPIPKEGIE--ISVYYSIADDFSFNIFCGFPPMV
QVD33034 DsLJV    1052 FSWRDHGDYNVGHIVLWSEEAFTCD----IFWSAGDDFEIKNFLGPPPVL

AUE23905 TcIV     1248 -------LTSELPLGTL-------PSTLEDFILPEMEYANP---EGLLDG
ATG71380 DWV      1144 -------ILDQLPAPVV-------R-------------AVP---EGPIAK
YP_003622540 SBPV 1127 -------YCDETYSAAT-------PD-LAQYFEDEVTIAQP---EMMMSM
QVD33034 DsLJV    1098 APFSKFALSDNHPSSQMEDFSPRERPSLARSIYDRISTPLACAGAAQIPV

AUE23905 TcIV     1282 VKNGVRSIVS-----------ESVQTTVKQAISEEM-----AKLDPYKKQ
ATG71380 DWV      1165 IKNFF----------------HQTADEVREVQAAKMREDMGIVVQDVIGE
YP_003622540 SBPV 1160 CQGFVGSLIGHHLQRATTYGIESAKESIRDVVKDEFKTQIKPELDNLNKV
QVD33034 DsLJV    1148 IGTAVATGIGAYKLGTTIANIEDKVGETTNNVNNLI-----HNADDLVSM

AUE23905 TcIV     1316 ILEKL--------EVNVNESIFKIV----IECLHLSQSRSIQTICLVVIQ
ATG71380 DWV      1199 LSQAIPDLQ----QPEVQANVFSLV----SQLVHAIIGTSLKTVAWAIVS
YP_003622540 SBPV 1210 IGEAANSIGASFGDILPQQLIINAM----GQLMQVFSNPSPVALAIAIVT
QVD33034 DsLJV    1193 IKQKIST------GLNITNNIYTAVVNGIIDLLTNMGRWDFKAISLSILR

AUE23905 TcIV     1354 VLLELKFIIVDNILGIGQVLVRAVTN-WLSRTSGEVIEDVADVPVVSPEA
ATG71380 DWV      1241 IFVTLGLIGREMMHSVITVVKRLLEK-YHLATQPQESASSGTVISAVPEA
YP_003622540 SBPV 1256 FIGSIVTLSMELVSTLSDSLRIFLEKVWY-RYFHQATEQQAAGASAMPEG
QVD33034 DsLJV    1237 LIINTINVPLTSIASYIPEFQNLFRR--------SPSAQAEDSIEQISES
                                 (VP1-Non-structural proteins cleavage site)
AUE23905 TcIV     1403 LYTNFEVKDWGPFVGMLFTGVTTALSLAVAPPKSFDT--FSKFLSKQI-P
ATG71380 DWV      1289 --PNAEAEEASAWVSIIYNGVCNMLNVAAQKPKQFKD--WVKLATVDF-S
YP_003622540 SBPV 1305 FWDEADDKSLHGILGMIFSAICVSLGLSMAPPKQFPS--VMKGVKESL-N
QVD33034 DsLJV    1279 FWNEQNIKDA---FKLICSIVGTAIGAVLIAPSNLPTDIIKSILTAPFRT

AUE23905 TcIV     1450 TVAKNFVFISTFVNSIVDLAKRMIRWIMHKVYPQEAWYIDMRDDAGELKD
ATG71380 DWV      1335 NNCRGSNQVFVFFKNTFEVLKKMWGYIFCQSNPAARLLKAVNDEPEILKA
YP_003622540 SBPV 1352 TA----NASVTFFRNVVDAIKYMYTYCMGASDEEMRARIIIEREYPNLKH
QVD33034 DsLJV    1326 TTVVLANGILRFADITFNIIKQACLWVASYFDPRVRIVQELRLQSPVIND

AUE23905 TcIV     1500 WVDEVLYLCSLDMDTRLDEDGYLYDRVYACLLYGREMAARYCG--NSDPK
ATG71380 DWV      1385 WVKECLYLDDPKFRMRRAHDQEYIERVFAAHSYGQILLHDLTAEMNQSRN
YP_003622540 SBPV 1398 WCEEVIQLLDPRSRNIVEHDARQASRVFDACIYGAQILQENLD--KSMPG
QVD33034 DsLJV    1376 FMKESQILTNEINRSAMQLPAFRL-RYWVNVVKAYEIQRRVIDLPRNAVS

AUE23905 TcIV     1548 IHLFMKVYDKINDLYWKMTAAGRHPFVRKEPFCIWMYGQPGIGKSFLTEH
ATG71380 DWV      1435 LSVFTRVYDQISKLKTDLMEMGSNPYIRRECFTICMCGASGIGKSYLTDS
YP_003622540 SBPV 1446 GKVIYDLYTRIVKLRDDLIELGNHPDVRFEAFPVWIVGSAGIGKSYNTTE
QVD33034 DsLJV    1425 PVLVRMCADVIKHGKENMADLRCSP-VKYEPYVLVIHGPAGIGKSNLVTH

AUE23905 TcIV     1598 ISSQLLQHIK-YKMVGPKIFTVMPACEFWTGCKNQPVLCMDDAFSVSTGN
ATG71380 DWV      1485 LCSELLRASR-TPVTTGIKCVVNPLSDYWDQCDFQPVLCVDDMWSVETST
YP_003622540 SBPV 1496 LCKRALQSIN-YRTKESMIYWLALGQKYWNGIRNPPVVARDEAYAV-SGQ
```

Fatehi et al. 2023. Characterization of Iflavirus in the Red Flour Beetle, *Tribolium Castaneum* (Tenebrionidae; Coleoptera).

```
QVD33034 DsLJV    1474 LAKEMLAAIGLTRFQSDPVHIRSAGSKHWNTYSDQPVVVYDDWLNITSPE

AUE23905 TcIV     1647 TMERQLNTMYMVKSPVTLNPPMADLKDKHLRYNPEIFYINSNIAFLN---
ATG71380 DWV      1534 TLDKQLNMLFQVHSPIVLSPPKADLEGKKMRYNPEIFIYNTNKPFPR---
YP_003622540 SBPV 1544 FTEEEISVHLAMCSSCILNPPMAALSEKNKRINPLIYYMNANCAFPS---
QVD33034 DsLJV    1524 LVASTLNELYQLKSCATFVPEQAAIEDKKIKATPRLVILLCNNAYPDSVL

AUE23905 TcIV     1694 VPGLD-AAAIHRRRDILIEAAIRDEGKQ----------PRDYPREVVKVF
ATG71380 DWV      1581 FDRIA-MEAIYRRRNVLIECRASEEKKRGCKHCENDIPIAECSPKMLKDF
YP_003622540 SBPV 1591 IPEARHIGAIYRRRKILAEFDFTEEIKR---TYPNVLDASELPPNARINN
QVD33034 DsLJV    1574 TNMVSCKDAVYRRRELTVHCARKPEYEN--------RNLREMSDEESLSM

AUE23905 TcIV     1733 GHLKFRTHKDPRVPSTEDNMWSEWMSYEELITIMKTQYRTFYDQEKENYD
ATG71380 DWV      1630 HHIKFRYAHDVC---NSETTWSEWMTYNEFLEWITPVYMANRRKANESFK
YP_003622540 SBPV 1638 NHLRIRVAHDPK---NVNTTWSEWMSFDEFSNHFCRKFQEHMEAERVNFR
QVD33034 DsLJV    1616 AHLLFRQDKGF----DSMNLSKKAVGYPEFKKFAIESFKEYDNQERRNVR

AUE23905 TcIV     1783 DRLATYHVIEDGQSEDQQRDDNNHIYSIGEISRNRHQRAID--EYTRAYN
ATG71380 DWV      1677 MRVDEMQMLRMDEP---------------LEGDNILNKYVE-------VN
YP_003622540 SBPV 1685 RRMDAAYAL---DPDYVPGSNLNYVGDYELPLQTLHERYIYERELAREYL
QVD33034 DsLJV    1662 QRLDKLMGYFSTTPCDIRDPFAVFYEGCAFATQDDMPLRSEMLEHEIRQV

AUE23905 TcIV     1831 ECLYVKMRGSFERMYE-----WMSDLN--MCQRIANYVNPMIVRVNT---
ATG71380 DWV      1705 QRLVEEMKAFKERTL------WSDLHR--VGAEISASVKKA---------
YP_003622540 SBPV 1732 ANIENPVNEIEEDGGF-----WSNVKR--L--------------------
QVD33034 DsLJV    1712 AGVIETARSDNEALNLSVHNPWSEEVRAQMIAEAACLLSVGTAAIGGLYY

AUE23905 TcIV     1870 -IANAGVPEMDLEDEQPCGIS--TLSASYPELRPHPVNSQSSSSTGRRSS
ATG71380 DWV      1737 -LPTISITEKLPHWTVQCGIAKPEMDHAYEVMSSYAAGMNAEIE-AHEQV
YP_003622540 SBPV 1754 -YENLTTGAADTEMDVP-GPSTPSEPPSKRVLRQFLVDHCKFKEDAAALL
QVD33034 DsLJV    1762 CLRKLGSYNASEGLEAQCNICLETTKIHYVCGASYSMLQTRSLEGVHKFC

AUE23905 TcIV     1918 THGSYSVNSSQPKSFREMQDTVSSEDQYFSIAPPKF--TTYFSDFKDGEL
ATG71380 DWV      1786 RXSSVECQYAEPQGLRRLDDEGPTIDEELMGDTEFA--SQAIERLVDE--
YP_003622540 SBPV 1803 TRGLGVCSEQDVRTFALPPEYQFMKDREFVSAASIY--CDTMSCLPNSVC
QVD33034 DsLJV    1812 RTCVNRVQSVGPWSCAVCRNNA---EPVLVMSQSMYESINVLSRLHKASN

AUE23905 TcIV     1966 IVNETQENRLHIFETS----------LECILKDRKIGQKDIDE------I
ATG71380 DWV      1831 ------GYITGEQKKY--IATW------CSKRREHIADFDLVWTDNLRVL
YP_003622540 SBPV 1851 CSYRRIGSAMNPRSKI--GSNW----FVLPKNYEDLGGKDAIRSYCYWWI
QVD33034 DsLJV    1859 WATNVIGNFINEFDRIVGVKNWGMFLLGCSYISSINTRVRLNGGDNYPYL

AUE23905 TcIV     2000 LKY-------------VQRGFGDIVLSEKE-----------NLGIFYHTR
ATG71380 DWV      1868 SAY-------------VHERSSSTRLSTDDVKLYK------TISMLHQKY
YP_003622540 SBPV 1895 RCY-------------QLRSFKKI-LKKENYVEWA------DRMLHLIGC
QVD33034 DsLJV    1909 MIYNASTAILSSVCFPQLYRFSRLTPATRTYIDLARTKLIDNIPSFSPEA

AUE23905 TcIV     2026 RAA-------ERNPREFWRRPWNFWKCVYYGL--------------KMGD
ATG71380 DWV      1899 DTT-------ECTKCQHWYAPLT-------DI--------------YVDD
YP_003622540 SBPV 1925 EPV-------NNYEVELRRQLWKIRGASESEIIDMLKELDAEARAHNMSD
QVD33034 DsLJV    1959 EAILARILEYNNSVSTNRFVPEATRTALERDITELVRHRLGDNFTRYIKP

AUE23905 TcIV     2055 TSGTQAIYTLREDWR-----DCVHMVSVDTPKNMSYDLFK----------
ATG71380 DWV      1921 K---------KLFWN-----QKEKKTLIDVRKLSKEDVTV----------
YP_003622540 SBPV 1968 LMAGKELPRITLMWLLTDLTDNDTSVFCDHCKVFATYM------------
QVD33034 DsLJV    2009 I---KGLFFTEEIATLSANIEQYNVALLANPNEWNEELYNSLIARIPPTA

AUE23905 TcIV     2089 ---EEGHHNWYMN---GIKVPH-----------CRGGKC-----------
ATG71380 DWV      1946 ---QSKLINLSVP--------------------C-GEVC-----------
YP_003622540 SBPV 2005 ---RDLSVLEYVPRYGIIRYPGNFGCMKTIPAECQCEDS-----------
```

Fatehi et al. 2023. Characterization of Iflavirus in the Red Flour Beetle, *Tribolium Castaneum* (Tenebrionidae; Coleoptera).

```
QVD33034 DsLJV    2056 QNDSDEEDDWSMPLRIGARRVNHFSVGLQNAQDYYPYTCDVTKWTGVAEF

AUE23905 TcIV     2111 ---IMDAPFFKALWHWAWLNNHTDQLWQYRKNELRYLPAFFNFDPSGRE-
ATG71380 DWV      1961 ---VLHSKYFNYLFHKAWL--LENPTWRLIYNG--SKKGMPEYFMNCVD-
YP_003622540 SBPV 2041 ---IFNNVLFKNAMRILWDHDHVGPEFTEFQDS-----QTNPFVMQEHNN
QVD33034 DsLJV    2106 NTICPHKHFVEHKEHILYD--KGNYFWNSVQGEVPIKDGWCSNYPECPMA

AUE23905 TcIV     2158 LKKELDDYFKSFTGRIVMWCKNTLGPMAVKVLKYVA--------------
ATG71380 DWV      2003 -EISLDSKFGKVKVWLQAIIDKYLTRPVKMIRDFLFKW-----WPQVAYV
YP_003622540 SBPV 2084 LRNETRSLLSRIWDWAKDWWKTTVVPFVGAILTFLYEH-----WAK----
QVD33034 DsLJV    2154 PGNREPHLLAYFEQHKNLWFGIFINKPPHVQKRFIEQLVPRFAWPQWIMT

AUE23905 TcIV     2193 ----VAGVCVGVACSMMW-GIKTLARLFGFNICDDAAVLLTAAATPKLHL
ATG71380 DWV      2048 LS--LLGII----------GITAYEMRNPKPTSEELADHYVNRHCSP-DF
YP_003622540 SBPV 2124 ----ILSIILG--CVVL--YATFTSAKSPTDACMAAAKVSVPVMAGAVGT
QVD33034 DsLJV    2204 EQNDIENPILE--TSSWWDYLNVHPMLVNILKVAAVGTVTIGILAGSYSL

AUE23905 TcIV     2239 KAGDFCIAEGA-YNIPSKIVKRPDVKV--MSKMPEMAPQQYSNIMAAINR
ATG71380 DWV      2085 WSPGMATPQGLKYSEAVTA-RAPRIHRLPVTTKPQGSTQQVDAAVNKILQ
YP_003622540 SBPV 2167 WS----SPEGSVYQAGERV---FKASNAPKPANRESVSDQMHITEQKVIN
QVD33034 DsLJV    2252 FRWLFPTPESQIIPSGDSVIRHFRARAQQITRVPASQSEFMDVIVDKINN

AUE23905 TcIV     2286 NTVIITAEYFNEKGS-LCSMKARALGLEANRVIMIRHYHDEFTAMPPSTR
ATG71380 DWV      2134 NMVYIGVVFPKVPGSKWRDINFRCLMLHNRQCLMLRHYLESTAAFPEGTK
YP_003622540 SBPV 2210 NTCFIICKW--QDGKDTKFLRARCLAIKGRDIIVIKHYLQEFKSRPNPTY
QVD33034 DsLJV    2302 NYFVIIVV--ENQGNERHLIGC---GVKQRLGIMPKHYYEYICKKKKEEG

AUE23905 TcIV     2335 YYVSLVTNNQLRPRVEINYRECRC----DAFYCK---DPKYGTNFFMLYM
ATG71380 DWV      2184 YYFKYIHNQETRMSGDISGIEIDLLNLPRLYYGGLAGEESFDSNIVLVTM
YP_003622540 SBPV 2258 MFSYKINNSMANTYIDSSIIDNAY-----IYKIN---NSSAFSNIALIKL
QVD33034 DsLJV    2347 VQFFLAKPHLIKQRVQLAIDEADF-------------TYSATADICLYQL

AUE23905 TcIV     2378 PPHIPLFRNIKNLIPTAESH----QYCGRLG-----HLYAFGDATYADLA
ATG71380 DWV      2234 PNRIPECKSIIKFIASHNEHMRA-QNDGVLVTGDHTQLLAFENNNKTPIS
YP_003622540 SBPV 2300 PKHVPMFKDISKSIVTQGDHANVGHFCSIVS-----QQYDEHPVVRSQVP
QVD33034 DsLJV    2384 PASWNMFRDITKYMSLDADLQKKMANNGVIV-----KPPLKSNNYTSVIP

AUE23905 TcIV     2419 FNFEGEYRIQQW-GNAPGMVCMNAYTYSKHKPGLCGSVLISEGL--PSTP
ATG71380 DWV      2283 INADGLY----------EVILQGVYTYPYHGDGVCGSILLSRNL---QRP
YP_003622540 SBPV 2345 VTWKQHLVIAGD-RHVEQIIMDKCYEYNVRGFGMCGSALVSPGVCCGNGG
QVD33034 DsLJV    2429 VDIKGYVKNQFIMGENGGFNSQHCLKYTFSQKGACGSMVLKDQS---QRP

AUE23905 TcIV     2466 LIGMHVAG-ANGK---GYAEPLFREMF------DMLSPLEIVDVAP-LDV
ATG71380 DWV      2320 IIGIHVAG-TEGLHGFGVAEPLVHEMFTGKAIESEREPYDRVYELP-LRE
YP_003622540 SBPV 2394 VIGLHVAG-EKGS---GFSEPIFREMF-----EPVIEKSDPVVSLPNLRS
QVD33034 DsLJV    2476 IVAMHIAGIGEGVSGIGYGVILTDEMF-AEFQCEVSGKCAEHEEPPYVEE

AUE23905 TcIV     2505 AEAKPEVSL--ETVVFEEGTCHPKMKHVETGKTAYIPSEIAG-VFPITYE
ATG71380 DWV      2368 LD-ESDIGL--DTDLYPIGRVDAKLAHAQSPSTGIKKTLIHG-TFDVRTE
YP_003622540 SBPV 2435 VS-ESNVQL--DHNLILYGCVDEKMSHKESGKSKIIPSLVHGEIYPVATE
QVD33034 DsLJV    2525 ID-TAKMHLPEDAVVGYVGVLPNNLKPFSPFKSKIKPSLIQD-LLPWTTT

AUE23905 TcIV     2552 PNP--LSPKDPRLPKGSNPIKDGCEKHGM-PVRPFPARMLERASYDLVQK
ATG71380 DWV      2414 PNP--MSSRDPRIAP-HDPLKLGCEKHGM-PCSPFNRKHLELATNHLKEK
YP_003622540 SBPV 2482 PNP--LRPGDPRQPPGSHPLRDGCAKHGLGMVHPFPQEDLEQVNNDARNV
QVD33034 DsLJV    2573 KAPAILSQKDERYTHTISPLVAGCAKHGY-LTANFTTSQLHKVR-DLRFE

AUE23905 TcIV     2599 FKACVVPVRAKVGVLSEQDAICGNVNVPHFESLEWNSSPGYGYADLKRSL
ATG71380 DWV      2460 LVSVVKP-INGCKIRSLQDAVCGVPGLDGFDSISWNTSAGFPLSSLKPP-
YP_003622540 SBPV 2530 LLNEVKNPLCEMRLLTLQEQVCGSTSIPHCESVNWNSSEGFPLCNRRPA-
```

Fatehi et al. 2023. Characterization of Iflavirus in the Red Flour Beetle, *Tribolium Castaneum* (Tenebrionidae; Coleoptera).

```
QVD33034 DsLJV     2621 QLRRMKPGISPCNRLTVEEAIIGFAGIEFYDPLHLDTSAGWPLCTSKKT-

AUE23905 TcIV      2649 GNPSGKKFLFDLDETPDG-YVLKGKHDRLTQIMNEKHEQRKKGIVPATVF
ATG71380 DWV       2507 -GTSGKRWLFDIELQDSGCYLLRGMRPELEIQLSTTQLMRKKGIKPHTIF
YP_003622540 SBPV 2578 -GVTGKKWLFDMDETAEG-YVLKNIDPQLALMLKTNKDLRNRNIVCPPIY
QVD33034 DsLJV     2669 ---LKKDWC-DIERDESGVLISCKLHPEVANQIESEMNKRRRGIVPFTVF

AUE23905 TcIV      2698 VDCLKDTTIPKEKCSVRGKTRIFSISPVDFTIQFKQYCGDFLAAYTKARF
ATG71380 DWV       2557 TDCLKDTCLPVEKCRIPGKTRIFSISPVQFTIPFRQYYLDFMASYRAARL
YP_003622540 SBPV 2627 IDCLKDYRLPPEKCCIPGKTRIFSIAPIQTTLEIREYMGLFLSGYKSATV
QVD33034 DsLJV     2716 VDTLKDEKKPLAKIHKLGGTRVFCASPFAYTVAMRQNFLHMCAAYSTYRW

AUE23905 TcIV      2748 RAEHAIGINVDSEEWTGLYNHVVTKGYNFITGDYSNFGPGLSLEVANAAF
ATG71380 DWV       2607 NAEHGIGIDVNSLEWTNLATSLSKYGTHIVTGDYKNFGPGLDSDVAASAF
YP_003622540 SBPV 2677 MGQHGIGINPDSYDWTRLANYLHEVGDNIVTGDYANFGPCVSSQIVYSCI
QVD33034 DsLJV     2766 ELKHAVGINLQGPQCTELVSRLLRVGHNIVTIDYSNFGPGYNAGVAEQAA

AUE23905 TcIV      2798 EIIVEW--YRYHGASEEHLRIL-RVMKCEILCARHLCLNVFYSVCCGIPS
ATG71380 DWV       2657 EIIIDWVLHYTEEDNKDEMKRVMWTMAQEILAPSHLCRDLVYRVPCGIPS
YP_003622540 SBPV 2727 DDIIYW--HKINGATEDHCRHLELLLKYSILLPLHLCDNCVYQSLNGIAS
QVD33034 DsLJV     2816 QNFKKWTLSNVAGVNEAELDCL----LEEGLNSLHCMNNVLYRQQGGSPS

AUE23905 TcIV      2845 GSPITTPLNSIVNSLYLRVMWMRMFNNHPDYFSLESFNAHVNIVTYGDDV
ATG71380 DWV       2707 GSPITDILNTISNGLLIRLAWLGITD-----LPLSEFSQNVVLVCYGDDL
YP_003622540 SBPV 2775 GSPITAELNSEVGKKYIKLAFLGICRQLNYKYSLNDFNKHCRVVVYGDDL
QVD33034 DsLJV     2862 GSPITVIINSEVNIMYIMLAWDALVKGEGKW---RDFEEQVCMYVYGDDL

AUE23905 TcIV      2895 IGCVSDEVRDLYNIASLSENFADFNITFTDARKTGVIVPYITDIREVWFL
ATG71380 DWV       2752 IMNVSDNMIDKFNAVTIGKFFSQYKMEFTDQDKSGNTVKWRT-LQTATFL
YP_003622540 SBPV 2825 ILSVSDAFVSWFNLQSISEYLDNYGIRLTDVTKDGTIVKYRP-LADSSFL
QVD33034 DsLJV     2909 IMSVSDNYIEHFNAVTITEFFKKHKIVATNADKSTEIKPYET-IETATFL

AUE23905 TcIV      2945 SRCFIPHPTRPMIWLAGLKKDSIQNAANWIKKK-FCKRDSSLINSRMAIE
ATG71380 DWV       2801 KHGFLKHPTRP-VFLANLDKVSVEGTTNWTHARGLGRRTATIENAKQALE
YP_003622540 SBPV 2874 KRSFKPHPSRSGIYLAPIEPRSYQECTNWCHKQ-NDEIEATVEVLRASCV
QVD33034 DsLJV     2958 KCNFVPHPYRSGEWLAKLDIESVNDTPMWIKEP-IAFKEATELNAEAGVR

AUE23905 TcIV      2994 LSFGWGPEYYREVCETVRKAWVAKNEVLYVKTWEERDKEIFDFKCESSIS
ATG71380 DWV       2850 LAFGWGPEYFNYVRSTIKMAFDKLGIYEDLITWEEMDVRCYASA------
YP_003622540 SBPV 2923 LAYGRGPDEYNNHVNKIRRVCALKGLRFDPLTWAALDKENFG--------
QVD33034 DsLJV     3007 AAFGHGKEFFNNYRTNINIALNQIKSDPILLDWHDIDDSFYGEGSSYNVG

AUE23905 TcIV      3044 FFPVIEF
ATG71380 DWV       2893 -------
YP_003622540 SBPV 2964 -------
QVD33034 DsLJV     3057 Q------
```
