# Supplementary material for: Characterization of Iflavirus in the Red Flour Beetle, Tribolium castaneum (Coleoptera; Tenebrionidae)
Source: Insects. 2023 Feb 23;14(3):220. doi: 10.3390/insects14030220 (PMC10051554; doi:10.3390/insects14030220)
Supplement: Supplementary file 1 [file insects-14-00220-s001.zip › Supplementary data S5. Free-of-TcIV Biosamples.pdf]

Fatehi et al. 2023. Characterization of Iflavirus in the Red Flour Beetle, *Tribolium Castaneum* (Tenebrionidae; Coleoptera).

Supplementary data S5. Free-of-TcIV Biosamples

SRX accession #	Geographic Location	Strain
SRX8948418…SRX8948661	University of Zurich (GEO)	Cro1
SRX8710218…SRX8710245	University of Cologne	San Bernardino
SRX8370579…SRX7895778	University of Kentucky	Unknown
SRX6384970…SRX6385152	University of Zurich (GEO)	Cro1
SRX425536…SRX425562	Leiden University (GEO)	San Bernardino
SRX5329740…SRX5329748	USDA, Insect Control and Cotton Disease Research, TX	Unknown
SRX4809500…SRX4809503	University of Kentucky	Unknown
SRX4782863-SRX4782864	German Center for Cancer Research (GEO)	GA-2
SRX4666274…SRX4666290	Texas A&M University (GEO)	GA-2
SRX4509171…SRX4509176	Anyang Institute of Technology	Unknown
SRX3238141…SRX3238156	Miami University (GEO)	Unknown
SRX2745603…SRX2944938	University of Kentucky	GA-2
SRX2741222…SRX2741229	University of Kentucky	QTC279 (pyrethriod resistant strain)
SRX2609558…SRX2609560	University Hospital Erlangen	Unknown
SRX1647966…SRX1669666	University of Cologne, Inst. for developmental Biol	San Bernadino | cultivar:AG Roth
SRX1396587…SRX1396874	University of Greifswald	Unknown
SRX878244…SRX878666	University of Greifswald	Unknown
SRX757105…SRX792460	California Institute of Technology (GEO)	Unknown
SRX501817…SRX501822	Georg-August-University of Goettingen	Unknown
SRX465141…SRX465165	Georg-August-University of Goettingen	San Bernardino
SRX021963…SRX022010	Emory University	Unknown
